# Undergraduate pediatrics clerkship student must-see list: prioritization and agreement between students and faculty in the UAE

**DOI:** 10.3389/fmed.2026.1876306

**Published:** 2026-07-24

**Authors:** Hamed Al Ahbabi, Ali Al Nuaimi, Khalfan Al-Shamsi, Ali Al-Afeefi, Abdulla Al Arwani, Ahmed Alsuwaidi, Elhadi H. Aburawi, S. Ayhan Çalışkan, Awad Al Essa, Imran Zafar, Sami Shaban, Mohi Eldin Magzoub

**Affiliations:** College of Medicine and Health Sciences, United Arab Emirates University, AlAin, United Arab Emirates

**Keywords:** curriculum prioritization, decision study, generalizability theory, ICC, must-see conditions, pediatrics clerkship, stakeholder agreement, survey methodology

## Abstract

**Background:**

Ensuring reliable exposure to core pediatric problems during the clerkship is challenging when case-mix varies across clinical sites. A data-informed, stakeholder-aligned “must-see” list can focus bedside encounters and guide supplemental teaching.

**Objective:**

To identify prioritized pediatric illnesses mapped to common clinical presentations, quantify agreement between students and faculty/physicians, and estimate the number of student raters needed for reliable curriculum monitoring.

**Design, setting, and participants:**

UAE-based cross-sectional survey of undergraduate medical students who completed the pediatrics clerkship and faculty/physicians involved in undergraduate pediatric teaching.

**Methods:**

Illnesses nested within clinical presentation sections were rated on a 5-point priority scale (5 = must see, 1 = must not see). Means ± SEM were summarized by illness and subgroup (students/doctors), illnesses were ranked, and group alignment was examined using Pearson r (95% CI via Fisher z), Spearman rho, and two-way ICCs based on illness-level subgroup means. Benjamini-Hochberg false discovery rate adjustment was applied to per-presentation tests. A generalizability-theory decision study (D-study) projected reliability (G-coefficient) across different numbers of student raters.

**Results:**

Forty-nine usable responses were analyzed (39 students, 10 faculty/physicians). Highest-rated items included asthma, bronchiolitis, pneumonia, gastroenteritis, febrile seizure, iron deficiency anemia, and heart murmur. Students’ and doctors’ illness-level means were strongly aligned (Pearson r = 0.863, 95% CI 0.794–0.910, *p* < 0.001; Spearman rho = 0.867, 95% CI 0.798–0.914, *p* < 0.001; *n* = 81). Agreement was good to excellent for subgroup means [ICC(2,1) = 0.920 (0.841–0.958); ICC(3,1) = 0.932 (0.862–0.963)]. The D-study indicated that 10 student raters were sufficient to reach G > =0.80 in these data (projected G = 0.949).

**Conclusion:**

Students and faculty/physicians showed strong alignment on a practical, presentation-based core of pediatric “must-see” illnesses. Programs can prioritize real-patient exposure for the highest-ranked problems, use simulation or structured cases when exposure is limited, and monitor exposure with low-burden periodic surveys.

## Introduction

Clinical clerkships are the bridge between preclinical learning and supervised patient care. Among these rotations, pediatrics poses distinct challenges: children present with illnesses, developmental conditions, and psychosocial contexts that differ substantially from adult medicine. For many undergraduates, the pediatric clerkship is their only sustained exposure to child health. Ensuring that graduates can recognize and manage common pediatric problems is therefore a core curricular priority.

To safeguard essential exposure, many programs articulate lists of “must-see” diagnoses, conditions that every medical student should encounter during pediatric training (1–4). These lists are intended to align clinical experience with curricular intent. However, exposure is shaped by local case-mix and institutional setting (e.g., tertiary centers versus community hospitals), so students’ real encounters can diverge from expectations. When that happens, graduates may be underprepared for common presentations such as bronchiolitis, asthma exacerbations, or dehydration.

Prior literature has described competency frameworks and learning outcomes (5), but less is known about whether student experience and faculty priorities converge around the same core of pediatric diagnoses. In the UAE, undergraduate medical education is guided by institutional curricula, accreditation expectations, and the EmiratesMEDs national competency framework, which articulates graduate competencies, entrustable professional activities, skills, and clinical presentations for medical programs (6). However, a specialty-specific undergraduate pediatrics “must-see” illness list for clerkship encounters has not been clearly delineated in the published UAE literature. Addressing this gap is essential for designing clerkships that are realistic, equitable across sites, and defensible to learners and accrediting bodies.

This UAE-based cross-sectional survey addressed the following research question: Which pediatric illnesses, mapped to common clinical presentations, should be prioritized for undergraduate clerkship exposure, and to what extent do students and faculty/physicians agree on those priorities? Findings are intended to inform prioritization of clinical exposure, targeted supplementation (e.g., simulation), and iterative curriculum design across clerkship sites.

This work is situated within published undergraduate pediatrics curricula that delineate core conditions and presentations for clerkships (1, 2) and evidence showing that student clinical exposure can vary across pediatric clerkship sites (3, 7–9).

## Objectives

- Establish a prioritized core list of “must-see” pediatric diagnoses mapped to common clinical presentations.
- Compare importance ratings between medical students and faculty/physicians and quantify agreement (correlations and ICCs).
- Identify presentations/diagnoses with high alignment versus mismatch to guide clerkship scheduling, logbook monitoring, simulation, and supplemental teaching.

## Methods

### Study design and setting

A UAE-based descriptive cross-sectional survey was conducted during two consecutive academic years. The study compared importance ratings for pediatric illnesses between undergraduate medical students who had completed the pediatric clerkship and faculty/physicians involved in undergraduate pediatric teaching. The term “prioritization” is used rather than “consensus” because a formal Delphi, nominal-group, or structured consensus-panel method was not used.

### Participants and recruitment

Two respondent groups were invited through the institutional email distribution lists of a single UAE undergraduate medical program: (1) students who completed the pediatric clerkship within the preceding two academic years and (2) faculty/physicians, including consultants, attendings, and academic pediatric teaching staff, engaged in undergraduate teaching. Participation was voluntary and anonymous. Because invitations were distributed through group email lists and the number of eligible recipients was not retained in the analytic dataset, a response rate could not be calculated. Institution/site, year of study, clerkship setting, and years of teaching experience were not collected in a form suitable for analysis. Respondents with no usable illness ratings were excluded listwise; otherwise, available ratings were analyzed using pairwise deletion.

### Survey instrument and content

A structured questionnaire administered through an institutional web-based survey platform captured importance ratings for illnesses nested under common pediatric clinical presentations (e.g., cough/wheeze, fever, dehydration). For each presentation, respondents rated specific illnesses using a 5-point priority scale: 5 = “must see,” 4 = “important to see,” 3 = “optional/nice to see,” 2 = “low priority,” and 1 = “must not see.” The preliminary list was derived from international frameworks and published pediatric clerkship exposure literature (1–4, 6) and was reviewed locally by the investigator team for relevance to UAE undergraduate pediatric teaching. The final list contained 81 presentation-illness pairs; some illnesses were intentionally retained under more than one presentation when clinically appropriate. Formal content-validity indices, cognitive interviewing, and pilot testing were not performed and are acknowledged as limitations. Reporting was reviewed against the CHERRIES checklist for internet survey studies (10). The item list and response scale are provided in Supplementary Table 1.

### Data management and preprocessing

Survey data were exported to a spreadsheet and cleaned before analysis. Duplicate illness labels were disambiguated by presentation (e.g., “Asthma [Cough and wheeze]”), textual responses were mapped to numeric scores, and the data were reshaped to one record per respondent, presentation, illness, and rating. Invalid values were set to missing; no imputation was performed.

### Outcomes

The primary outcome was the mean importance rating for each Presentation × Illness pair. Secondary outcomes included median, standard deviation (SD), standard error of the mean (SEM), top-two-box (≥4), and “must-see” (=5) percentages. Subgroup outcomes summarized the same metrics separately for students and doctors.

### Statistical analysis

All analyses used two-sided alpha = 0.05. For each Presentation x Illness pair, *N* (available ratings), mean, median, SD, SEM, top-two-box percentage (rating > = 4), and must-see percentage (rating = 5) were summarized, and illnesses were ranked within each presentation by mean rating. Mann–Whitney U tests were used as exploratory item-level comparisons; *p*-values in descriptive tables are unadjusted and were interpreted cautiously given the number of comparisons and the small faculty/physician group. Using illness-level subgroup means, alignment between students and doctors was quantified with Pearson’s r (95% CI via Fisher z) and Spearman’s rho. Benjamini-Hochberg false discovery rate (FDR) adjustment was applied to per-presentation *p*-values (reported as q-values); per-presentation correlations were computed only where at least three illnesses had non-missing means for both subgroups. Agreement was estimated from illness-level subgroup means using two-way random-effects ICC(2,1) (absolute agreement, single-measure) and two-way mixed-effects ICC(3,1) (consistency, single-measure), with average-measure ICCs (k = 2). Individual-response variance-component models were explored but were not interpreted when convergence or component estimates were unstable. Analyses were conducted in Python (pandas, scipy, statsmodels); figures were generated with matplotlib.

### Sample size and decision study (generalizability theory)

The number of students required to obtain reliable illness-level estimates was estimated using a Generalizability Theory (G-theory) decision study (D-study). This was a *post hoc* reliability-planning exercise rather than an *a priori* power calculation for recruitment. The measurement object was the illness; students were the random facet (raters). Variance components were obtained from a variance-components model fitted to individual ratings (random intercepts for Illness and Respondent).

For a single-facet crossed design (Illness × Student), the relative G-coefficient for the mean rating based on *n* students is:

$$G(n)=\sigma^{2}\_\text{Illness}/[\sigma^{2}\_\text{Illness}+(\begin{array}{l} \sigma^{2}\_\text{Illness}\times \text{Student}\\ +\sigma^{2}\_\text{residual} \end{array})/n]$$

Using the estimated variance components, G(n) was projected for *n* = 10 to 200 students, and the smallest *n* achieving reliability thresholds of G > = 0.80 (primary) and G > = 0.70 (lenient) was identified. Projection within clinical presentations was planned when sufficient data were available. As a sensitivity check, the Spearman-Brown prophecy was applied to the single-measure ICC to obtain an alternative n: ICC_n = (*n* × ICC_1)/[1 + (*n* − 1) × ICC_1], solved for the target reliability.

These projections quantify how many student respondents are needed for stable illness-level monitoring in future cohorts. Analyses followed established practices, including FDR control for multiple testing (11), selection and reporting of intraclass correlation coefficients per Koo and Li (12), and the use of generalizability theory with D-study projections to inform reliability and required sample sizes (13, 14).

### Ethics

This study involved human participants and was approved by the relevant Institutional Review Board [UAE University ERSC_2025_8089, 5/8/2025]. Completion of the anonymous online survey indicated informed consent. No identifiable personal data were collected.

## Results

The analytic sample included 49 respondents with usable ratings: 39 students and 10 faculty/physicians. No additional demographic variables were available for analysis. The highest mean ratings were observed for asthma, bronchiolitis, pneumonia, gastroenteritis, febrile seizure, iron deficiency anemia, and heart murmur (Supplementary Table 2; Table 1).

**Table 1:** Mean ± SEM for the top- and bottom-ranked illnesses (doctor, student, and all; pairwise available ratings, *n* up to 49).

Group	Clinical presentation	Illness	Doctors (*n* = 10) Mean ± SEM	Students (*n* up to 39) Mean ± SEM	Mann–Whitney *p*-value	ALL (*n* up to 49) Mean ± SEM
Top 10	Cough and wheeze	Asthma	5.00 ± 0.000	4.95 ± 0.036	0.469	4.96 ± 0.029
	Cough and wheeze	Bronchiolitis	5.00 ± 0.000	4.87 ± 0.066	0.296	4.90 ± 0.053
	Cough and wheeze	Pneumonia	4.90 ± 0.100	4.90 ± 0.061	0.834	4.90 ± 0.053
	Seizures	Febrile seizure	5.00 ± 0.000	4.76 ± 0.088	0.168	4.81 ± 0.072
	Vomiting	Gastroenteritis	4.90 ± 0.100	4.74 ± 0.082	0.340	4.77 ± 0.068
	Abdominal pain	Gastroenteritis	4.80 ± 0.133	4.71 ± 0.084	0.661	4.73 ± 0.071
	Diarrhea	Gastroenteritis	5.00 ± 0.000	4.66 ± 0.094	0.056	4.73 ± 0.077
	Anemia	Iron deficiency anemia	4.90 ± 0.100	4.66 ± 0.109	0.316	4.71 ± 0.089
	Cough and wheeze	Viral upper respiratory tract infection	4.90 ± 0.100	4.64 ± 0.101	0.221	4.69 ± 0.084
	Heart murmur	Innocent and pathologic murmur	4.90 ± 0.100	4.55 ± 0.105	0.101	4.63 ± 0.087
Bottom 10	Abdominal mass	Stool	4.10 ± 0.233	3.47 ± 0.188	0.115	3.60 ± 0.160
	White pupillary reflex	Retinoblastoma	3.50 ± 0.307	3.63 ± 0.148	0.690	3.60 ± 0.132
	Limp or extremity pain	Slipped capital femoral epiphysis	3.40 ± 0.267	3.58 ± 0.139	0.412	3.54 ± 0.123
	Rash	Scabies	3.70 ± 0.260	3.42 ± 0.134	0.395	3.48 ± 0.119
	Proteinuria	Orthostatic proteinuria	3.50 ± 0.307	3.39 ± 0.149	0.809	3.42 ± 0.133
	Limp or extremity pain	Legg-Calve-Perthes disease	3.40 ± 0.267	3.39 ± 0.134	0.837	3.40 ± 0.118
	Limp or extremity pain	Nursemaid elbow	3.60 ± 0.267	3.32 ± 0.147	0.377	3.38 ± 0.128
	Abdominal pain	Pelvic inflammatory disease	3.67 ± 0.333	3.24 ± 0.166	0.258	3.32 ± 0.149
	Limp or extremity pain	Osgood Schlatter disease	3.30 ± 0.300	3.18 ± 0.140	0.820	3.21 ± 0.126
	Abdominal mass	Pregnancy	3.00 ± 0.422	3.08 ± 0.224	0.856	3.06 ± 0.196

The complete item-level table is provided in Supplementary Table 2 to improve main-text readability. Table 1 highlights the extremes to contextualize the full list. The top 10 illnesses were uniformly near the ceiling for both faculty/physicians and students, supporting their designation as priority exposure targets. In contrast, the bottom 10 entries were consistently lower across both groups. The largest faculty-student mean differences were seen for occult bacteremia, urticaria, postnasal drip, and appendicitis, with faculty/physician ratings higher than student ratings; these items may warrant discussion in clerkship orientation and assessment blueprints.

**Figure 1:**
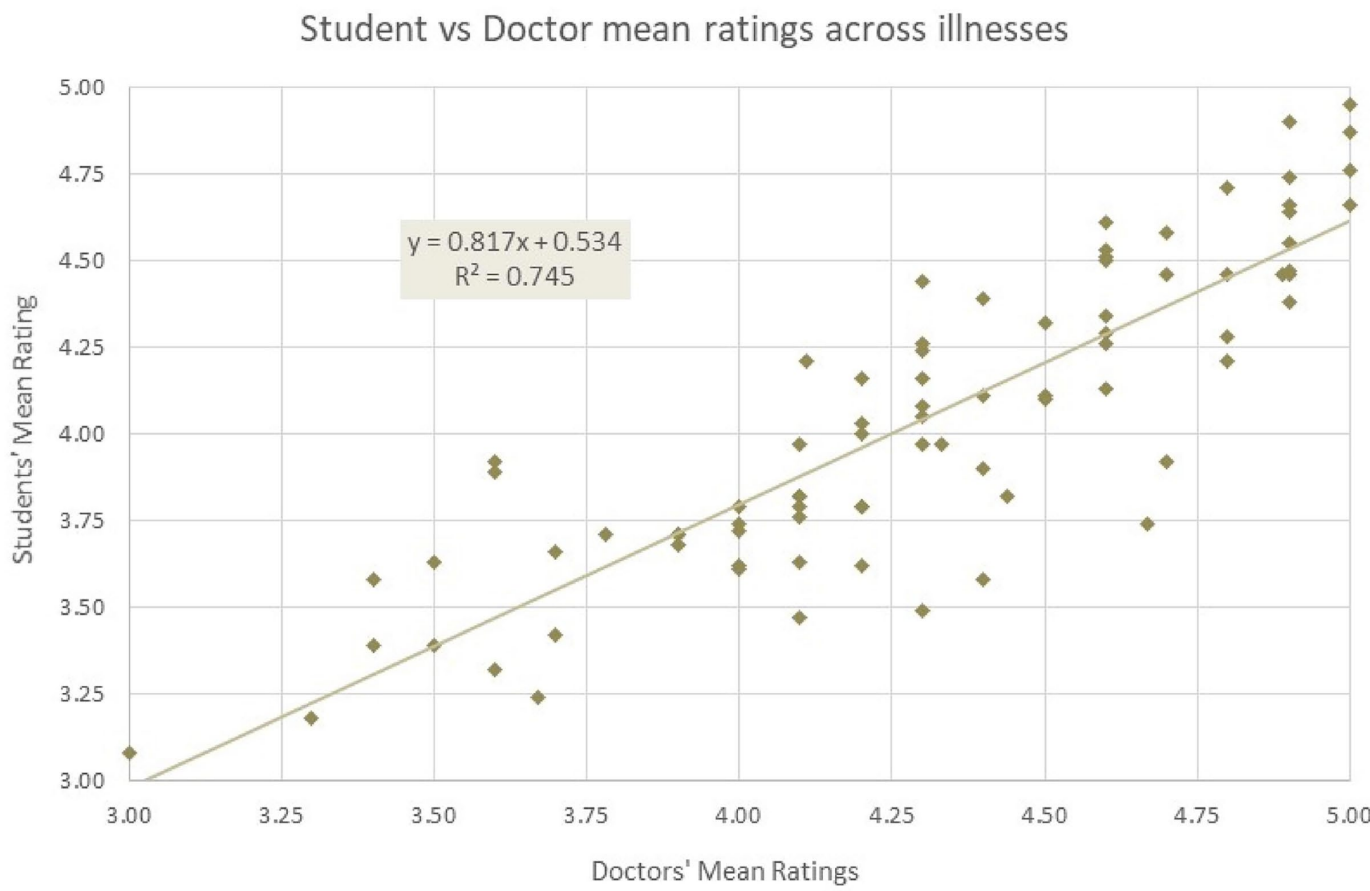
Students vs. doctors mean ratings across illnesses (scatter plot of doctors’ mean ratings on the *x*-axis and students’ mean ratings on the *y*-axis for each illness; the diagonal reference line indicates perfect agreement).

Figure 1 illustrates a scatter plot showing a strong linear relationship between students’ and doctors’ mean ratings with tight clustering around the identity line. This indicates high alignment without systematic over- or under-rating by either group. A few outliers reflect presentations where emphasis differs, but these do not materially affect the overall agreement.

Correlation analysis shown in Table 2 confirmed strong agreement between groups at the illness level: Pearson’s r was high with a narrow 95% confidence interval, and Spearman’s rho similarly indicated near-monotonic alignment. After controlling for multiple testing across presentations using FDR, only a small number of presentations showed statistically significant correlations, which is consistent with the limited number of illnesses per presentation; therefore, the overall illness-level signal is emphasized.

**Table 2:** Correlation between students’ and doctors’ illness-level means (*n* = 81).

Statistic	Value	95% CI
Pearson correlation *r*	0.863	[0.794, 0.910]
Pearson *p*-value	<0.001*	–
Spearman correlation *ρ*	0.867	[0.798, 0.914]
Spearman *p*-value	<0.001*	–

The bold value and * indicate *p* < 0.01.

Agreement indices in Tables 3, 4 derived from subgroup means indicated good-to-excellent agreement for both absolute agreement ICC(2,1) and consistency ICC(3,1), with higher values for average-measure ICCs (k = 2), as expected. Individual-response variance-component ICCs could not be estimated stably and are therefore not interpreted.

**Table 3:** Agreement indices (ICCs) estimated from illness-level subgroup means (two-way models) and exploratory individual-response variance-components models.

Model	ICC type	Estimate	95% CI
Illness-level subgroup means	ICC(2,1) absolute (single)	0.920	[0.841, 0.958]
Illness-level subgroup means	ICC(2,k) absolute (avg of 2)	0.958	–
Illness-level subgroup means	ICC(3,1) consistency (single)	0.932	[0.862, 0.963]
Illness-level subgroup means	ICC(3,k) consistency (avg of 2)	0.965	–
Individual responses	ICC(A,1) absolute (single)	N/A	–
Individual responses	ICC(C,1) consistency (single)	N/A	–

**Table 4:** Decision study (G-theory) projection for required student sample size.

Target reliability	Minimum *n* students	Projected G(n)
G ≥ 0.70	10	0.949
G ≥ 0.80	10	0.949

Using students’ individual ratings in a one-facet (Illness x Student) random-effects model, variance components for Illness and residual error were estimated. The reliability (G-coefficient) of illness-level means was then projected as the number of student raters increased. The minimum number of student raters was 10 for both thresholds (G > = 0.70 and G > = 0.80), with projected G = 0.949 at *n* = 10. The 39 student respondents in the present sample therefore exceeded the D-study monitoring target. These values should be interpreted as reliability projections for item-level monitoring rather than as evidence of population representativeness.

## Discussion

In this UAE-based prioritization and agreement study, faculty/physicians and medical students rated “must-see” pediatric illnesses within common clinical presentations in a broadly similar way. Across the full item set, the faculty-student correlation was high, indicating strong concordance in rank-order preferences. Agreement statistics based on illness-level subgroup means were good to excellent. Some individual illnesses showed residual disagreement, an expected feature when judgments reflect different roles, clinical exposure, perceived prevalence, and assessment expectations. High agreement may also partly reflect a ceiling effect because many items were rated between 4 and 5; this supports the perceived importance of the core list but limits discrimination among highly rated conditions.

The findings align with established undergraduate pediatrics curricula that emphasize a core set of acute and chronic conditions and age-based presentations that all trainees should encounter during clerkship (1, 2). They are also consistent with the EmiratesMEDs emphasis on national graduate competencies, clinical presentations, and alignment of curricula with local health-system needs (6). Multi-site evaluations have shown that student outcomes can be comparable across diverse training locations when clerkship objectives, assessment methods, and faculty development are standardized (7, 8). Earlier work likewise reported that students can achieve satisfactory performance in pediatric clerkships across varied clinical settings (9). Together, these observations support the feasibility of defining a shared set of high-yield illnesses and presentations while acknowledging that real-patient exposure will vary by site and season.

Methodologically, two complementary approaches were applied. First, Pearson correlations captured alignment in relative priorities (rank order), while intraclass correlation coefficients reflected absolute agreement, both recommended for multi-rater educational data (12). Second, generalizability (G) theory decomposed observed variation into illness, rater, and residual components, enabling decision (D) studies to project how many respondents are needed to reach target reliability (13, 14). To address multiplicity in item-wise comparisons, the false discovery rate was controlled using the Benjamini-Hochberg procedure, a standard approach that maintains power while limiting expected false positives (11).

Educational implications include a pragmatic core list of illnesses per presentation that both groups rate as high priority, targeted dialogue for illnesses with larger faculty-student gaps, and resourcing guidance from D-study projections for future curriculum reviews (13, 14). Implementation could include minimum expected encounters or equivalent learning activities for the highest-ranked conditions, logbook monitoring to detect missed exposure, simulation or structured case-based learning for uncommon but high-consequence conditions, and alignment with OSCE stations, mini-CEX encounters, workplace-based assessment, and competency-based evaluation. The lower mean ratings for the bottom 10 entries may indicate that guaranteed real-patient exposure is less feasible or less central during a short clerkship. Lower-ranked items should therefore be viewed as lower priority for guaranteed bedside exposure within a limited clerkship, not as conditions of lower clinical importance; these topics can be covered through structured cases, skills labs, simulation, or self-study when real-patient encounters are limited.

Limitations include the small and imbalanced sample (39 students and 10 faculty/physicians), the single-program UAE context with affiliated pediatric teaching sites but without analyzable site-level distribution, inability to calculate a response rate because the invitation denominator was not retained, and absence of detailed participant characteristics such as institution, year of study, clerkship setting, academic role, and teaching experience. Anonymous voluntary participation and broad distribution through institutional lists were used to reduce social desirability and coercion; however, self-selection and nonresponse bias cannot be excluded. The survey list was adapted from published frameworks and local investigator review, but formal content-validity indices, cognitive interviewing, pilot testing, and exploratory or confirmatory factor analysis were not undertaken. The instrument was designed as a presentation-based priority list rather than a latent construct scale, which limits the appropriateness of EFA/CFA in this first study. The original five-category scale may compress variance and produce ceiling effects; the category 1 wording (“must not see”) was not formally validated and may have been understood as a recommendation to avoid seeing the condition, whereas the authors intended it to indicate the lowest priority for guaranteed clerkship exposure. Future work should sample multiple schools and clerkship sites with documented denominators, collect participant characteristics, include additional stakeholders such as curriculum leaders, residents, patients, and parents, integrate patient-encounter logs to anchor priority ratings to authentic exposure, and link priorities to assessment blueprints and outcomes.

## Conclusion

Faculty/physicians and students largely agree on which illnesses should be prioritized within common pediatric presentations, consistent with published undergraduate pediatrics curricula and competency frameworks (1–6). Where differences exist, they are interpretable and useful for continuous quality improvement. The analytic framework—correlations, ICCs, FDR-aware comparisons, and G-/D-studies—offers a transparent and reproducible template for other clerkships to align learning priorities across stakeholders (11–14).

## Data Availability

The raw data supporting the conclusions of this article will be made available by the authors, without undue reservation.
