# Supplementary material for: Undergraduate pediatrics clerkship student must-see list: prioritization and agreement between students and faculty in the UAE
Source: Front Med (Lausanne). 2026 Jul 24;13:1876306. doi: 10.3389/fmed.2026.1876306 (PMC13448782; doi:10.3389/fmed.2026.1876306)
Supplement: Supplementary file 1 [file Table_1.DOCX]

**Supplementary Materials**

**Supplementary Table 1: Survey instrument item list**

*Respondents rated each illness within its clinical presentation using the following priority scale:* ***5 = must see; 4 = important to see; 3 = optional/nice to see; 2 = low priority; 1 = not necessary/lowest priority.*** *The item list below shows the presentation-by-illness structure used for the survey. Some illnesses appear under more than one presentation when clinically appropriate.*

| **Clinical presentation** | **Illness items** |
| --- | --- |
| Abdominal mass | Hydronephrosis; Malignancy; Pregnancy; Stool |
| Abdominal pain | Appendicitis; Constipation/encopresis; Gastroenteritis; HSP; Intussusception; Pelvic inflammatory disease; Urinary tract infection/ pyelonephritis |
| Anemia | Iron deficiency anemia; Sickle cell anemia; Thalassemia |
| Bruising, Petechiae, purpura | Trauma, ITP; Vasculitis; Viral infections |
| Cough and wheeze | Asthma; Bronchiolitis; Croup; Pneumonia; Viral upper respiratory tract infection |
| Diarrhea | Gastroenteritis |
| Fever and rash | Group A streptococcal infection; Kawasaki disease; Meningococcemia; Viral exanthem |
| Fever without a focus | Bacteremia/sepsis; Meningitis; Occult bacteremia; Urinary tract infection; Viral illness |
| Headache | Meningitis; Tension headache; Trauma |
| Heart murmur | Innocent and pathologic murmur |
| Hematuria | Glomerulonephritis, UTI; Trauma |
| Limp or extremity pain | Developmental dysplasia of the hip; Fracture; Legg-Calve-Perthes disease; Nursemaid elbow; Osgood Schlatter disease; Osteomyelitis; Septic arthritis; Slipped capital femoral epiphysis; Transient synovitis |
| Lymphadenopathy | Malignancy (leukemia); Mononucleosis; Viral illnesses, bacterial adenitis |
| Otalgia | Otitis externa; Otitis media; Otitis media with effusion |
| Positive Mantoux skin test | latent and active tuberculosis |
| Proteinuria | Nephrotic syndrome; Orthostatic proteinuria |
| Rash | Atopic, contact and seborrhea dermatitis; Cellulitis, candida; Impetigo; Scabies; Urticaria; Viral enanthem |
| Red eye | Conjunctivitis |
| Rhinorrhea | Allergic rhinitis; Sinusitis; Viral URI |
| Seizures | Febrile seizure; Sepsis |
| Sore Throat | Group A streptococcal pharyngitis; Mononucleosis; Postnasal drip; Viral upper respiratory tract infection |
| Splenomegaly, hepatomegaly | Hepatitis; Malignancy; Mononucleosis |
| Vomiting | Gastroenteritis; Gastroesophageal reflux; Pyloric stenosis; UTI/pyelonephritis |
| White pupillary reflex | Cataracts; Retinoblastoma |

**Supplementary Table 2: Mean ± SEM for all clinical presentations and relevant pediatric illnesses**

*Values are based on available ratings for each item. Pairwise deletion was used for item-level summaries; therefore, denominators may vary slightly across items.*

| **Clinical Presentation** | **Illness** | **Doctors**  **(n=10)**  **Mean ± SEM** | **Students**  **(n=39)**  **Mean ± SEM** | **Mann-Whitney p-value** | **ALL**  **(n=49)**  **Mean ± SEM** |
| --- | --- | --- | --- | --- | --- |
| Abdominal mass | Hydronephrosis | 3.70 ± 0.335 | 3.66 ± 0.147 | 0.925 | 3.67 ± 0.134 |
| Abdominal mass | Malignancy | 4.10 ± 0.277 | 3.79 ± 0.156 | 0.360 | 3.85 ± 0.136 |
| Abdominal mass | Pregnancy | 3.00 ± 0.422 | 3.08 ± 0.224 | 0.856 | 3.06 ± 0.196 |
| Abdominal mass | Stool | 4.10 ± 0.233 | 3.47 ± 0.188 | 0.115 | 3.60 ± 0.160 |
| Abdominal pain | Appendicitis | 4.70 ± 0.213 | 3.92 ± 0.153 | 0.018* | 4.08 ± 0.136 |
| Abdominal pain | Constipation/encopresis | 4.50 ± 0.224 | 4.10 ± 0.141 | 0.202 | 4.18 ± 0.123 |
| Abdominal pain | Gastroenteritis | 4.80 ± 0.133 | 4.71 ± 0.084 | 0.661 | 4.73 ± 0.071 |
| Abdominal pain | HSP | 4.00 ± 0.211 | 3.62 ± 0.114 | 0.122 | 3.69 ± 0.102 |
| Abdominal pain | Intussusception | 4.00 ± 0.258 | 3.74 ± 0.126 | 0.352 | 3.80 ± 0.113 |
| Abdominal pain | Pelvic inflammatory disease | 3.67 ± 0.333 | 3.24 ± 0.166 | 0.258 | 3.32 ± 0.149 |
| Abdominal pain | Urinary tract infection/ pyelonephritis | 4.50 ± 0.167 | 4.32 ± 0.126 | 0.623 | 4.35 ± 0.105 |
| Anemia | Iron deficiency anemia | 4.90 ± 0.100 | 4.66 ± 0.109 | 0.316 | 4.71 ± 0.089 |
| Anemia | Sickle cell anemia | 4.60 ± 0.221 | 4.61 ± 0.110 | 0.962 | 4.60 ± 0.098 |
| Anemia | Thalassemia | 4.70 ± 0.153 | 4.58 ± 0.104 | 0.701 | 4.60 ± 0.088 |
| Bruising, Petechiae, purpura | Trauma, ITP | 4.50 ± 0.224 | 4.11 ± 0.129 | 0.157 | 4.19 ± 0.114 |
| Bruising, Petechiae, purpura | Vasculitis | 3.90 ± 0.277 | 3.71 ± 0.155 | 0.592 | 3.75 ± 0.135 |
| Bruising, Petechiae, purpura | Viral infections | 4.30 ± 0.260 | 4.24 ± 0.138 | 0.869 | 4.25 ± 0.121 |
| Cough and wheeze | Asthma | 5.00 ± 0.000 | 4.95 ± 0.036 | 0.469 | 4.96 ± 0.029 |
| Cough and wheeze | Bronchiolitis | 5.00 ± 0.000 | 4.87 ± 0.066 | 0.296 | 4.90 ± 0.053 |
| Cough and wheeze | Croup | 4.60 ± 0.221 | 4.51 ± 0.109 | 0.663 | 4.53 ± 0.097 |
| Cough and wheeze | Pneumonia | 4.90 ± 0.100 | 4.90 ± 0.061 | 0.834 | 4.90 ± 0.053 |
| Cough and wheeze | Viral upper respiratory tract infection | 4.90 ± 0.100 | 4.64 ± 0.101 | 0.221 | 4.69 ± 0.084 |
| Diarrhea | Gastroenteritis | 5.00 ± 0.000 | 4.66 ± 0.094 | 0.056 | 4.73 ± 0.077 |
| Fever and rash | Group A streptococcal infection | 4.30 ± 0.213 | 4.44 ± 0.109 | 0.508 | 4.41 ± 0.096 |
| Fever and rash | Kawasaki disease | 4.30 ± 0.153 | 3.97 ± 0.135 | 0.297 | 4.04 ± 0.113 |
| Fever and rash | Meningococcemia | 4.10 ± 0.233 | 3.82 ± 0.146 | 0.409 | 3.88 ± 0.126 |
| Fever and rash | Viral exanthem | 4.40 ± 0.163 | 3.90 ± 0.146 | 0.118 | 4.00 ± 0.124 |
| Fever without a focus | Bacteremia/sepsis | 4.80 ± 0.133 | 4.21 ± 0.123 | 0.023* | 4.33 ± 0.107 |
| Fever without a focus | Meningitis | 4.80 ± 0.133 | 4.28 ± 0.122 | 0.036* | 4.39 ± 0.104 |
| Fever without a focus | Occult bacteremia | 4.67 ± 0.236 | 3.74 ± 0.136 | 0.005* | 3.92 ± 0.129 |
| Fever without a focus | Urinary tract infection | 4.90 ± 0.100 | 4.38 ± 0.130 | 0.047* | 4.49 ± 0.110 |
| Fever without a focus | Viral illness | 4.90 ± 0.100 | 4.46 ± 0.132 | 0.102 | 4.55 ± 0.109 |
| Headache | Meningitis | 4.60 ± 0.163 | 4.53 ± 0.098 | 0.814 | 4.54 ± 0.084 |
| Headache | Tension headache | 3.90 ± 0.233 | 3.71 ± 0.160 | 0.686 | 3.75 ± 0.135 |
| Headache | Trauma | 4.20 ± 0.200 | 4.00 ± 0.160 | 0.696 | 4.04 ± 0.133 |
| Heart murmur | Innocent and pathologic murmur | 4.90 ± 0.100 | 4.55 ± 0.105 | 0.101 | 4.63 ± 0.087 |
| Hematuria | Glomerulonephritis, UTI | 4.40 ± 0.267 | 4.39 ± 0.116 | 0.865 | 4.40 ± 0.106 |
| Hematuria | Trauma | 3.60 ± 0.221 | 3.92 ± 0.143 | 0.276 | 3.85 ± 0.123 |
| Limp or extremity pain | Developmental dysplasia of the hip | 4.00 ± 0.258 | 3.79 ± 0.132 | 0.482 | 3.83 ± 0.117 |
| Limp or extremity pain | Fracture | 4.10 ± 0.180 | 3.82 ± 0.150 | 0.314 | 3.88 ± 0.125 |
| Limp or extremity pain | Legg-Calve-Perthes disease | 3.40 ± 0.267 | 3.39 ± 0.134 | 0.837 | 3.40 ± 0.118 |
| Limp or extremity pain | Nursemaid elbow | 3.60 ± 0.267 | 3.32 ± 0.147 | 0.377 | 3.38 ± 0.128 |
| Limp or extremity pain | Osgood Schlatter disease | 3.30 ± 0.300 | 3.18 ± 0.140 | 0.820 | 3.21 ± 0.126 |
| Limp or extremity pain | Osteomyelitis | 4.30 ± 0.260 | 4.05 ± 0.125 | 0.357 | 4.10 ± 0.112 |
| Limp or extremity pain | Septic arthritis | 4.30 ± 0.260 | 4.16 ± 0.144 | 0.683 | 4.19 ± 0.125 |
| Limp or extremity pain | Slipped capital femoral epiphysis | 3.40 ± 0.267 | 3.58 ± 0.139 | 0.412 | 3.54 ± 0.123 |
| Limp or extremity pain | Transient synovitis | 4.10 ± 0.233 | 3.76 ± 0.166 | 0.328 | 3.83 ± 0.141 |
| Lymphadenopathy | Malignancy (leukemia) | 4.20 ± 0.249 | 4.16 ± 0.128 | 0.913 | 4.17 ± 0.113 |
| Lymphadenopathy | Mononucleosis | 4.10 ± 0.233 | 3.63 ± 0.109 | 0.074 | 3.73 ± 0.102 |
| Lymphadenopathy | Viral illnesses, bacterial adenitis | 4.60 ± 0.163 | 4.29 ± 0.119 | 0.252 | 4.35 ± 0.101 |
| Otalgia | Otitis externa | 4.00 ± 0.333 | 3.72 ± 0.151 | 0.452 | 3.77 ± 0.137 |
| Otalgia | Otitis media | 4.89 ± 0.111 | 4.46 ± 0.121 | 0.106 | 4.54 ± 0.103 |
| Otalgia | Otitis media with effusion | 4.11 ± 0.200 | 4.21 ± 0.137 | 0.498 | 4.19 ± 0.116 |
| Positive Mantoux skin test | latent and active tuberculosis | 4.00 ± 0.211 | 3.61 ± 0.153 | 0.199 | 3.69 ± 0.130 |
| Proteinuria | Nephrotic syndrome | 4.60 ± 0.267 | 4.50 ± 0.105 | 0.391 | 4.52 ± 0.099 |
| Proteinuria | Orthostatic proteinuria | 3.50 ± 0.307 | 3.39 ± 0.149 | 0.809 | 3.42 ± 0.133 |
| Rash | Atopic, contact and seborrhea dermatitis | 4.44 ± 0.176 | 3.82 ± 0.150 | 0.058 | 3.94 ± 0.130 |
| Rash | Cellulitis, candida | 4.10 ± 0.233 | 3.97 ± 0.122 | 0.699 | 4.00 ± 0.107 |
| Rash | Impetigo | 4.20 ± 0.200 | 3.79 ± 0.120 | 0.106 | 3.88 ± 0.106 |
| Rash | Scabies | 3.70 ± 0.260 | 3.42 ± 0.134 | 0.395 | 3.48 ± 0.119 |
| Rash | Urticaria | 4.40 ± 0.163 | 3.58 ± 0.134 | 0.004* | 3.75 ± 0.121 |
| Rash | Viral enanthem | 4.20 ± 0.133 | 3.79 ± 0.147 | 0.161 | 3.87 ± 0.121 |
| Red eye | Conjunctivitis | 4.60 ± 0.163 | 4.34 ± 0.121 | 0.383 | 4.40 ± 0.102 |
| Rhinorrhea | Allergic rhinitis | 4.60 ± 0.163 | 4.13 ± 0.143 | 0.141 | 4.22 ± 0.121 |
| Rhinorrhea | Sinusitis | 4.33 ± 0.167 | 3.97 ± 0.130 | 0.231 | 4.04 ± 0.111 |
| Rhinorrhea | Viral URI | 4.70 ± 0.153 | 4.46 ± 0.115 | 0.375 | 4.51 ± 0.097 |
| Seizures | Febrile seizure | 5.00 ± 0.000 | 4.76 ± 0.088 | 0.168 | 4.81 ± 0.072 |
| Seizures | Sepsis | 4.90 ± 0.100 | 4.47 ± 0.111 | 0.058 | 4.56 ± 0.094 |
| Sore Throat | Group A streptococcal pharyngitis | 4.80 ± 0.133 | 4.46 ± 0.109 | 0.150 | 4.53 ± 0.093 |
| Sore Throat | Mononucleosis | 4.20 ± 0.200 | 3.62 ± 0.125 | 0.034* | 3.73 ± 0.112 |
| Sore Throat | Postnasal drip | 4.30 ± 0.213 | 3.49 ± 0.160 | 0.019* | 3.65 ± 0.142 |
| Sore Throat | Viral upper respiratory tract infection | 4.60 ± 0.163 | 4.26 ± 0.146 | 0.366 | 4.33 ± 0.122 |
| Splenomegaly, hepatomegaly | Hepatitis | 4.30 ± 0.213 | 4.08 ± 0.133 | 0.465 | 4.13 ± 0.114 |
| Splenomegaly, hepatomegaly | Malignancy | 4.20 ± 0.291 | 4.03 ± 0.139 | 0.469 | 4.06 ± 0.124 |
| Splenomegaly, hepatomegaly | Mononucleosis | 3.90 ± 0.277 | 3.68 ± 0.114 | 0.517 | 3.73 ± 0.106 |
| Vomiting | Gastroenteritis | 4.90 ± 0.100 | 4.74 ± 0.082 | 0.340 | 4.77 ± 0.068 |
| Vomiting | Gastroesophageal reflux | 4.40 ± 0.221 | 4.11 ± 0.135 | 0.321 | 4.17 ± 0.117 |
| Vomiting | Pyloric stenosis | 3.60 ± 0.306 | 3.89 ± 0.135 | 0.362 | 3.83 ± 0.124 |
| Vomiting | UTI/pyelonephritis | 4.30 ± 0.213 | 4.26 ± 0.134 | 0.945 | 4.27 ± 0.114 |
| White pupillary reflex | Cataracts | 3.78 ± 0.324 | 3.71 ± 0.146 | 0.754 | 3.72 ± 0.132 |
| White pupillary reflex | Retinoblastoma | 3.50 ± 0.307 | 3.63 ± 0.148 | 0.690 | 3.60 ± 0.132 |

* Significant at α<0.05
